# Exploring the relationship and shared mechanisms of major depressive disorder and diabetic kidney disease: a comprehensive clinical and genetic analysis

**DOI:** 10.3389/fpsyt.2025.1546733

**Published:** 2025-09-29

**Authors:** Huan-xi Liu, Ting-ting Wang, You-di Cheng, Chun-hui Qu, Shun-kang Feng, Xiang-wen Wang, Xiao-hui Wu, Wen-xi Sun, Ping Sun

**Affiliations:** ^1^ Qingdao University, Qingdao Medical College, Qingdao, China; ^2^ Qingdao Mental Health Center, Qingdao, Shandong, China; ^3^ Clinical Research Center, Shanghai Mental Health Center, Shanghai Jiao Tong University School of Medicine, Shanghai, China; ^4^ Suzhou Guangji Hospital, Suzhou, Jiangsu, China; ^5^ Affiliated Guangji Hospital of Soochow University, Suzhou, Jiangsu, China

**Keywords:** major depressive disorder, diabetic kidney disease, NHANES, genetic correlation, transcriptomic analysis

## Abstract

**Introduction:**

Major depressive disorder (MDD) is a common comorbidity in diabetes mellitus (DM), while diabetic kidney disease (DKD) represents a severe complication of DM. However, the clinical and genetic associations between MDD and DKD remain unclear. This study aimed to investigate their shared biomarkers, molecular pathways, and immune features.

**Methods:**

We analyzed data from the National Health and Nutrition Examination Survey (NHANES, 2005–2018) to assess the association between MDD and DKD. Genetic correlation was evaluated using linkage disequilibrium score regression (LDSC), and causality was tested with Mendelian randomization (MR). Gene expression datasets were integrated to identify crosstalk genes, followed by protein–protein interaction (PPI) analysis to detect hub genes. Diagnostic performance was validated using least absolute shrinkage and selection operator (LASSO) regression and receiver operating characteristic (ROC) curves. Immune infiltration was assessed, and potential therapeutic compounds were predicted through connectivity map (cMAP) analysis and molecular docking.

**Results:**

Clinical analysis revealed a significant association between MDD and DKD (OR = 1.45, 95% CI: 1.28–1.64). LDSC indicated a significant genetic correlation (r = 0.2153, P = 0.008), although MR analysis did not support a causal relationship. A total of 83 crosstalk genes were identified, primarily enriched in inflammation and immune regulation pathways. PPI analysis highlighted eight hub genes, with CD163 and KLRB1 emerging as promising shared diagnostic biomarkers. Validation using LASSO and ROC confirmed their diagnostic potential. Immune infiltration analysis revealed shared immune cell alterations. Furthermore, cMAP analysis and molecular docking suggested rucaparib and levocetirizine as candidate therapeutic agents.

**Discussion:**

Our findings demonstrate a genetic and immunological link between MDD and DKD. CD163 and KLRB1 may serve as potential biomarkers and therapeutic targets, offering new insights into the shared mechanisms and treatment strategies for comorbid MDD and DKD.

## 1 Introduction

Diabetic kidney disease (DKD) is a clinical manifestation of the kidneys in diabetic patients characterized by proteinuria, hypertension, and a progressive decline in renal function. DKD is a frequent microvascular complication arising from diabetes mellitus (DM), affecting approximately 30% to 40% of DM patients (1, 2). Along with the increasing prevalence of DM globally, the incidence of DKD is also on the rise. DKD is a primary contributor to chronic kidney disease and renal failure, significantly impacting patients’ quality of life and prognosis, and it may even lead to death (2). Major depressive disorder (MDD) ranks among the prevalent mental disorders characterized by enduring feelings of sadness, reduced appetite, decreased interest in activities, hopelessness, sleep disorder, and even suicidal behavior in severe cases (3). The prevalence of MDD has rapidly increased worldwide in recent years, with more than 700,000 individuals committing suicide due to MDD each year, thereby imposing a heavy burden on individuals and society (4).

Compared to the general population, individuals with DM have twice the likelihood of experiencing depression and anxiety disorders. Diabetes-related complications, including DKD, are closely correlated with depression (5, 6). Cohort studies have shown that DKD patients with depression progress to end-stage renal disease at a rapid rate (7). Similarly, patients with DKD typically have more symptoms of depression and anxiety, often resulting in unfavorable clinical outcomes, such as accelerated renal function decline, increased hospitalizations, elevated mortality rates, and poor quality of life (8, 9). The pathophysiological mechanisms related to MDD include dysregulation of the hypothalamic–pituitary–adrenal-immune axis and activation of proinflammatory cytokines, which may lead to insulin resistance and heighten the risk of developing DM and its associated complications (10). A meta-analysis has indicated a bidirectional relationship between MDD and DKD, with DKD potentially predicting MDD and MDD serving as an indicator of DKD (11).

However, despite accumulating epidemiological evidence on the association between MDD and DKD, the underlying molecular and genetic mechanisms linking the two diseases remain largely unexplored. In particular, there is a lack of studies identifying key shared genes or pathways involved in this comorbidity. Further research is warranted to explore the relationship between MDD and DKD, particularly concerning cellular and molecular mechanisms. The present study employed bioinformatics techniques to identify genes involved in the crosstalk between MDD and DKD, revealing the potential mechanisms underlying the interactions between these two diseases and predicting small molecule compounds with therapeutic potential.

## 2 Materials and methods

**Figure 1:**
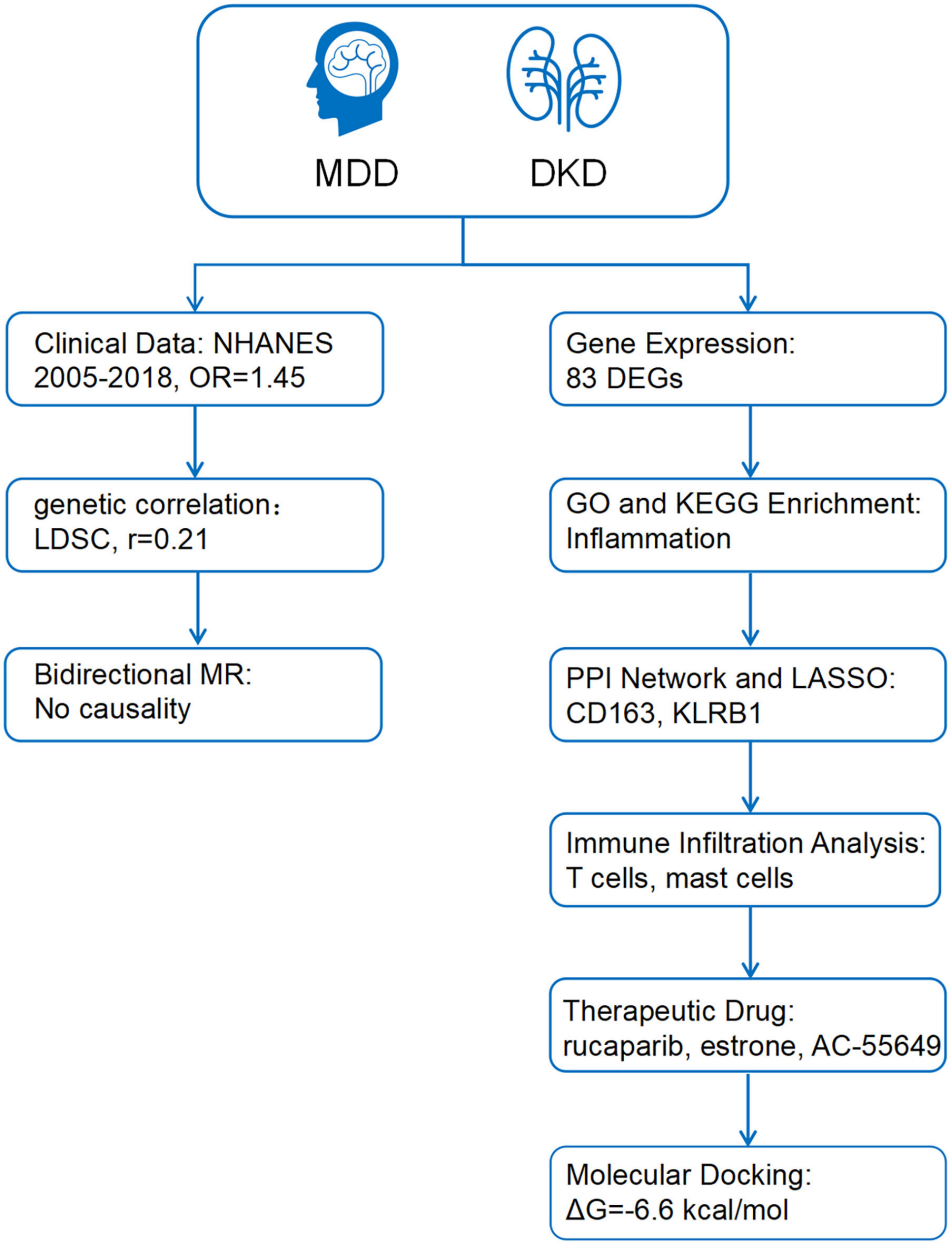
The working flow chart of this study. MDD, Major depressive disorder; DKD, diabetic kidney disease; NHANES, National Health and Nutrition Examination Survey; LDSC, Linkage Disequilibrium Score Regression; MR, Mendelian randomization; DEGs, differentially expressed genes; PPI, Protein-protein interaction; LASSO, least absolute shrinkage and selection operator.

This study utilized repeated cross-sectional data from the National Health and Nutrition Examination Survey (NHANES) cycles between 2005 and 2018, combined with genome-wide association study (GWAS) data, to investigate the genetic correlation and causal relationship between MDD and DKD using linkage disequilibrium score regression (LDSC) and bidirectional Mendelian randomization (MR). Differentially expressed genes were identified and subjected to functional enrichment, protein-protein interaction (PPI) network construction, and least absolute shrinkage and selection operator (LASSO) regression to select key biomarkers. Potential therapeutic drugs were screened via the Connectivity Map (cMAP) database and validated through molecular docking to explore drug-target interactions, aiming to elucidate shared mechanisms and therapeutic targets for both diseases (**Figure 1**).

### 2.1 Data collection and processing

The study utilized repeated cross-sectional data from NHANES cycles conducted between 2005 and 2018. NHANES is a nationwide survey that provides comprehensive health and nutrition data from a representative sample of the non-institutionalized U.S. population through complex, multistage sampling methods. The diagnostic criteria for diabetes were as follows: a) a previous diagnosis reported by a healthcare professional; b) fasting plasma glucose ≥7.0 mmol/L; c) glycated hemoglobin (HbA1c) ≥6.5%; d) 2-hour plasma glucose level ≥11.1 mmol/L during an Oral Glucose Tolerance Test (OGTT); or e) the use of diabetes medications or insulin (12, 13). According to the KDIGO 2021 Guidelines, CKD was defined as having a urinary albumin-to-creatinine ratio (UACR) > 30 mg/g and/or an estimated glomerular filtration rate (eGFR) < 60 mL/min/1.73 m² (14). DKD was defined as CKD combined with diabetes mellitus. Participants with a total PHQ-9 score of ≥ 10 were considered to have MDD. In addition, age, gender, race/ethnicity, education level, poverty income ratio (PIR), marital status, smoking status, body mass index (BMI), blood pressure, hypertension, high cholesterol, and coronary heart disease (CHD) were included as covariates. The final sample size for this study was 15,574 after systematic exclusion (**Supplementary Figure 1**).

The GWAS data required for LDSC and MR analyses were sourced from public databases (**Supplementary Table 1**). From these data, 157 genetic instruments were identified for assessing MDD and 10 for evaluating DKD.

Gene expression profiling datasets associated with MDD and DKD were obtained from the Gene Expression Omnibus (GEO) database (https://www.ncbi.nlm.nih.gov/geo/). The MDD dataset, GSE98793, which utilizes the GPL570 platform (Affymetrix Human Genome U133_Plus2.0), consists of 192 samples, including 128 samples from MDD patients and 64 samples from healthy control individuals. The DKD dataset, GSE30122, encompasses three datasets that are all based on the GPL570 platform (Affymetrix Human Genome U133A 2.0), and it comprises 69 samples, including 50 DKD patients and 19 healthy control individuals.

### 2.2 Statistical analysis

Due to the skewed distribution of the data, categorical variables are presented as frequencies (percentages), and continuous variables are presented as medians (interquartile ranges). The Chi-square test or Mann-Whitney U test was used to assess differences in DKD and MDD between exposed and unexposed groups. Logistic regression models were employed to calculate the odds ratios (ORs) for DKD and MDD. Subsequently, multivariable regression analysis was conducted to adjust for the effects of covariates, yielding adjusted odds ratios.

Data were weighted to produce accurate estimates that reflect the non-institutionalized civilian population of the United States. Statistical analyses were conducted using the Survey package in R software (version 4.2.3). A two-sided p <0.05 was considered statistically significant.

### 2.3 Genetic correlation analysis

LDSC analysis was performed to assess the genetic correlation between MDD and DKD using the software available at https://github.com/bulik/ldsc (15). Subsequently, bidirectional two-sample MR analysis was conducted with DKD and MDD as exposure and outcome variables. Sensitivity and heterogeneity tests were also conducted to validate the MR findings.

### 2.4 Analysis of differentially expressed genes

All operations were conducted in R (version 4.2.3). After preprocessing and normalizing the data, the limma package in R was used to identify DEGs. DEGs with a corrected p <0.05 and |log FC| ≥0.5 in the GSE30122 dataset and with a corrected p <0.05 and |log FC| ≥0.1 in the GSE98793 dataset were screened (16, 17). The more lenient threshold in the GSE98793 dataset was adopted to avoid overlooking potentially important DEGs with modest expression changes. Clustered heatmaps and volcano plots of the DEGs were generated using the pheatmap and ggplot2 packages in R, respectively. The ggVenn package in R was used to create Venn diagrams to identify genes involved in crosstalk between MDD and DKD for further analysis.

### 2.5 GO and KEGG enrichment analyses

Gene Ontology (GO) enrichment analysis is a structured, computerized approach aimed at elucidating the functions of genes and gene products, encompassing biological processes (BP), cellular components (CC), and molecular functions (MF). The Kyoto Encyclopedia of Genes and Genomes (KEGG) pathway analysis serves as a widely utilized enrichment tool to unveil biochemical mechanisms and functions (18). The identified crosstalk genes were subjected to GO and KEGG functional enrichment analyses, employing the clusterProfiler package in R. The ggplot2 and ggrepel packages in R were used to visualize the results.

### 2.6 Construction of the PPI network and identification of hub genes

The STRING database (https://string-db.org) is commonly utilized for constructing PPI networks (19). The screened crosstalk genes were imported into the STRING database to construct a PPI network, which had combined scores exceeding 0.4. The network was visualized using Cytoscape version 3.9.1, Cytoscape Consortium. Hub genes were identified by the Cytohubba plugin and MCODE algorithm.

### 2.7 Identification of biomarkers using LASSO analysis

LASSO analysis is a regression technique designed to enhance prediction accuracy by identifying variables with strong predictive power and low correlation from high-dimensional data (20). The glmnet package in R was used to perform LASSO regression, which identified the influential predictive factors among the hub genes that may serve as diagnostic biomarkers for MDD and DKD.

### 2.8 Expression levels and diagnostic value of candidate biomarkers

The ggplot2 package in R was used to create boxplots to assess biomarker expression levels (p <0.05). The pROC package in R was used to compute the area under the curve (AUC) of receiver operating characteristic (ROC) curves to assess the validity of potential shared diagnostic biomarkers in the GSE98793 and GSE30122 datasets.

### 2.9 Immune infiltration analysis

CIBERSORTx (https://cibersortx.stanford.edu/) is an online platform for immune infiltration analysis, and it was used to explore the differences in the distribution of immune cells between patients with both MDD and DKD and healthy individuals. Finally, Spearman rank correlation analysis was employed to assess the correlation between the expression levels of potential shared diagnostic biomarkers and the abundance of infiltrating immune cells, with a significance threshold set at p <0.05.

### 2.10 cMAP analysis and molecular docking

cMAP (https://clue.io/) is a gene expression profiling database that employs gene expression signature interventions to unveil connections among drugs, genes, and diseases, aiding in the screening of potential drug candidates (21). In the present study, co-upregulated DEGs related to MDD and DKD were uploaded to the cMAP database to identify potential therapeutic drugs. The top 10 drug candidates with the most significant negative scores were selected as potential therapeutics for MDD and DKD.

To evaluate the binding affinity between the aforementioned small-molecule drugs and biomarkers, molecular docking analysis was conducted. The three-dimensional structures of the target proteins were retrieved from the RCSB Protein Data Bank (https://www.rcsb.org/) , and PyMOL software (version 2.5.0) was used to remove water molecules, ligands, and other modifications (22, 23). The 3D structures of the small molecules were obtained from the PubChem database (https://pubchem.ncbi.nlm.nih.gov/) , followed by hydrogen addition and charge assignment. Finally, molecular docking was performed using AutoDock Vina (version 1.1.2), and binding sites with binding energies lower than –5.0 kcal/mol were considered to indicate stable interactions.

## 3 Results

### 3.1 Relationship between MDD and DKD

**Tables 1**, **2** present the baseline characteristics of the patients and the results of the logistic regression analysis for DKD and MDD. The results showed that MDD was significantly associated with an increased risk of DKD. In the univariate logistic regression analysis, the prevalence of MDD was higher in the DKD group than in the non-DKD group [OR = 1.45, 95% confidence interval (CI), 1.28-1.64]. The association remained significant after adjustment for covariates (adjusted OR = 1.24, 95% CI, 1.07-1.42).

**Table 1:** Baseline characteristics and OR of participants by DKD levels in NHANES (2005–2018).

Variables	Total (n = 15574)	Non-DKD (n = 11928)	DKD (n = 3646)	P value	OR (95%CI)	Adjusted OR (95%CI)
MDD				< 0.0001		
No	14203 (92.33%)	10968 (92.87%)	3235 (89.83%)		Reference	Reference
Yes	1371 (7.67%)	960 (7.13%)	411 (10.17%)		1.45 (1.28, 1.64)	1.24 (1.07, 1.42)
Age(years)	50.00 (37.00, 63.00)	48.00 (35.00, 59.00)	65.00 (53.00, 75.00)	< 0.0001	1.06 (1.06, 1.07)	1.05(1.04,1.05)
PIR	3.11 (1.57, 5.00)	3.30 (1.65, 5.00)	2.40 (1.30, 4.29)	< 0.0001	0.87 (0.85, 0.89)	0.89 (0.87, 0.92)
BMI(kg/m2)	28.70 (24.80, 33.50)	28.40 (24.59, 33.10)	30.20 (26.00, 35.50)	< 0.0001	1.03 (1.02, 1.03)	1.02 (1.02, 1.03)
ASBP(mmHg)	120.67 (111.33, 132.00)	119.33 (110.00, 129.33)	130.67 (116.67, 146.67)	< 0.0001	1.03 (1.03, 1.04)	1.01 (1.01, 1.01)
ADBP(mmHg)	70.67 (64.00, 77.33)	70.67 (64.00, 77.33)	68.67 (59.33, 77.33)	< 0.0001	0.99 (0.98, 0.99)	0.99 (0.99, 0.99)
Gender				0.0003		
Male	7711 (49.06%)	5910 (49.97%)	1801 (44.83%)		Reference	Reference
Female	7863 (50.94%)	6018 (50.03%)	1845 (55.17%)		1.01 (0.93, 1.08)	0.98 (0.89, 1.07)
Race				< 0.0001		
Mexican American	2208 (7.76%)	1739 (7.89%)	469 (7.15%)		Reference	Reference
Non-Hispanic White	6681 (68.82%)	5074 (69.08%)	1607 (67.62%)		1.17 (1.05, 1.32)	0.93 (0.81, 1.07)
Non-Hispanic Black	3259 (10.17%)	2281 (9.28%)	978 (14.32%)		1.59 (1.40, 1.80)	1.36 (1.18, 1.58)
Other Hispanic	1472 (5.35%)	1213 (5.63%)	259 (4.02%)		0.76 (0.65, 0.89)	0.68 (0.57, 0.82)
Other Race	1954 (7.90%)	1621 (13.59%)	333 (6.90%)		0.79 (0.67, 0.94)	1.04 (0.87, 1.24)
Education level				< 0.0001		
Below high school	3565 (14.32%)	2478 (12.92%)	1087 (20.84%)		Reference	Reference
High School or above	12009 (85.68%)	9450 (87.08%)	2559 (79.16%)		0.62 (0.57, 0.67)	0.96 (0.86, 1.06)
Marital status				< 0.0001		
No	5967 (33.99%)	4323 (32.58%)	1644 (40.57%)		Reference	Reference
Yes	9607 (66.01%)	7605 (67.42%)	2002 (59.43%)		0.69(0.64, 0.75)	0.83 (0.76, 0.91)
Smoke				0.0002		
No	8548 (54.82%)	6724 (55.73%)	1824 (50.58%)		Reference	Reference
Yes	7026 (45.18%)	5204 (44.27%)	1822 (49.42%)		1.29 (1.20, 1.39)	0.98 (0.90, 1.07)
Hyptersion				< 0.0001		
No	7883 (56.67%)	7037 (63.22%)	846 (26.13%)		Reference	Reference
Yes	7691 (43.33%)	4891 (36.78%)	2800 (73.87%)		4.76 (4.37, 5.18)	1.81 (1.63, 2.01)
High cholesterol level				< 0.0001		
No	9149 (60.63%)	7492 (63.63%)	1657 (46.62%)		Reference	Reference
Yes	6425 (39.37%)	4436 (36.37%)	1989 (53.38%)		2.03 (1.88, 2.19)	1.03 (0.95, 1.13)
CHD				< 0.0001		
No	14727 (95.30%)	11523 (96.81%)	3204 (88.25%)		Reference	Reference
Yes	847 (4.70%)	405 (3.19%)	442 (11.75%)		3.93 (3.41, 4.52)	1.78 (1.52, 2.07)

MDD, Major depressive disorder; DKD, diabetic kidney disease; PIR, poverty income ratio; BMI, body mass index; SBP, Systolic Blood Pressure; DBP, Diastolic Blood Pressure; CHD, coronary heart disease.

**Table 2:** Baseline characteristics and OR of participants by MDD levels in NHANES (2005–2018).

Variables	Total (n = 15574)	Non-MDD (n = 14203)	MDD (n = 1371)	P value	OR(95%CI)	Adjusted OR(95%CI)
DKD				< 0.0001		
No	11928 (82.35%)	10968 (82.83%)	960 (76.59%)		Reference	Reference
Yes	3646 (17.65%)	3235 (17.17%)	411 (23.41%)		1.45 (1.28, 1.64)	1.19 (1.03, 1.36)
Age(years)	50.00 (37.00, 63.00)	50.00 (37.00, 63.00)	51.00 (39.00, 62.00)	0.25	1.00 (1.00, 1.01)	0.99 (0.99, 1.00)
PIR	3.11 (1.57, 5.00)	3.25 (1.68, 5.00)	1.64 (0.91, 3.20)	< 0.0001	0.67 (0.64, 0.70)	0.73 (0.70, 0.77)
BMI(kg/m2)	28.70 (24.80, 33.50)	28.50 (24.80, 33.23)	30.90 (25.60, 36.00)	< 0.0001	1.04 (1.03, 1.05)	1.03 (1.02, 1.04)
ASBP(mmHg)	120.67 (111.33, 132.00)	120.67 (111.33, 132.00)	120.67 (111.33, 132.67)	0.41	1.00 (1.00, 1.00)	0.99 (0.99, 0.99)
ADBP(mmHg)	70.67 (64.00, 77.33)	70.67 (63.33, 77.33)	70.00 (64.00, 78.00)	0.15	1.00 (1.00, 1.01)	1.01 (1.01, 1.02)
Gender				< 0.0001		
Male	7711 (49.06%)	7180 (49.98%)	531 (37.96%)		Reference	Reference
Female	7863 (50.94%)	7023 (50.02%)	840 (62.04%)		1.61 (1.45, 1.82)	1.67 (1.47, 1.89)
Race				0.008		
Mexican American	2208 (7.76%)	2028 (7.86%)	180 (6.50%)		Reference	Reference
Non-Hispanic White	6681 (68.82%)	6069 (69.02%)	612 (66.39%)		1.14 (0.96, 1.35)	1.38 (1.14, 1.67)
Non-Hispanic Black	3259 (10.17%)	2964 (9.96%)	295 (12.68%)		1.12 (0.92, 1.36)	1.06 (0.86, 1.31)
Other Hispanic	1472 (5.35%)	1310 (5.24%)	162 (6.65%)		1.39 (1.11, 1.74)	1.48 (1.17, 1.86)
Other Race	1954 (7.90%)	1832 (7.92%)	122 (7.78%)		0.75 (0.59, 0.95)	1.18 (0.92, 1.52)
Education level				< 0.0001		
Below high school	3565 (14.32%)	3094 (13.49%)	471 (24.30%)		Reference	Reference
High School or above	12009 (85.68%)	11109 (86.51%)	900 (75.70%)		0.53 (0.47, 0.60)	0.71 (0.62, 0.81)
Marital status				< 0.0001		
No	5967 (33.99%)	5229 (32.69%)	738 (49.68%)		Reference	Reference
Yes	9607 (66.01%)	8974 (67.31%)	633 (50.32%)		0.50 (0.45, 0.56)	0.65 (0.57, 0.73)
Smoke				< 0.0001		
No	8548 (54.82%)	8011 (56.30%)	537 (37.06%)		Reference	Reference
Yes	7026 (45.18%)	6192 (43.70%)	834 (62.94%)		2.01 (1.79, 2.25)	1.89 (1.67, 2.13)
Hyptersion				< 0.0001		
No	7883 (56.67%)	7337 (57.61%)	546 (45.38%)		Reference	Reference
Yes	7691 (43.33%)	6866 (42.39%)	825 (54.62%)		1.61 (1.44, 1.81)	1.39 (1.20, 1.61)
High cholesterol level				< 0.0001		
No	9149 (60.63%)	8467 (61.24%)	682 (53.31%)		Reference	Reference
Yes	6425 (39.37%)	5736 (38.76%)	689 (46.69%)		1.49 (1.33, 1.67)	1.39 (1.22, 1.58)
CHD				0.0004		
No	14727 (95.30%)	13464 (95.54%)	739 (92.31%)		Reference	Reference
Yes	847 (4.70%)	1263 (4.46%)	108 (7.69%)		1.56 (1.26, 1.91)	1.33 (1.06, 1.67)

### 3.2 Genetic correlation

LDSC analysis revealed a significant genetic correlation between MDD and DKD, with a correlation coefficient of 0.2153 (p = 0.008) (**Supplementary Table 2**). Although the MR analysis indicated no causal relationship between the two diseases (**Supplementary Figure 2**), this result was supported by sensitivity tests (**Supplementary Table 3**).

### 3.3 Identification of crosstalk genes for MDD and DKD

**Figure 2:**
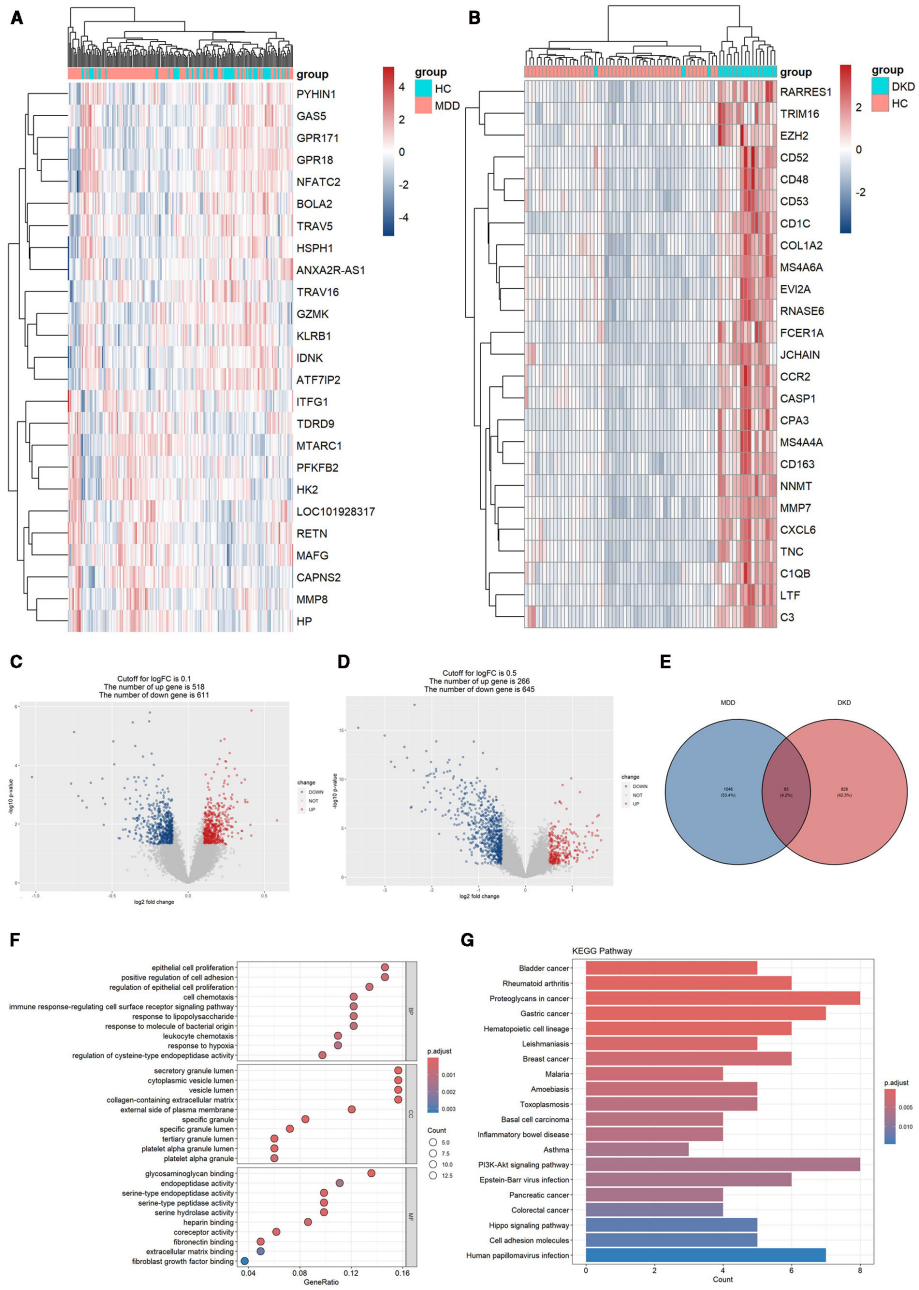
DEG expression in the two datasets and functional enrichment analyses of the crosstalk genes. **(A)** Heatmap of the top 25 DEGs in the GSE98793 dataset. **(B)** Heatmap of the top 25 DEGs in the GSE30122 dataset. **(C)** Volcano plot of DEGs in the GSE98793 dataset. **(D)** Volcano plot of DEGs in the GSE30122 dataset. **(E)** Identification of 83 genes related to crosstalk between the DEGs of MDD and DKD. **(F)** GO analysis of the crosstalk genes. **(G)** KEGG pathway enrichment analysis of the crosstalk gene.

After data preprocessing, a total of 1128 DEGs were identified from the GSE98793 dataset, including 518 upregulated genes and 611 downregulated genes (**Figures 2A, C**). From the GSE30122 dataset, a total of 828 DEGs were identified, encompassing 266 upregulated genes and 645 downregulated genes (**Figures 2B, D**). Altogether, 83 genes related to MDD and DKD crosstalk were identified by Venn diagrams (**Figure 2E**), of which 12 DEGs were commonly upregulated (ZNF91, TGFBR3, PCDH9, FGF9, CD83, COL4A3, P3H2, KANK3, ZBTB10, MID2, RABL3, and WNT10B).

### 3.4 GO and KEGG enrichment analyses of crosstalk genes

The 83 crosstalk genes were subjected to GO enrichment analysis, and a total of 374 GO terms were obtained, comprising 301 BP terms, 26 CC terms, and 47 MF terms (**Figure 2F**). Regarding the BP terms, the genes related to crosstalk were primarily enriched in epithelial cell proliferation (GO:0050673), positive regulation of cell adhesion (GO:0045785), regulation of epithelial cell proliferation (GO:0050678), cell chemotaxis (GO:0060326), and the immune response-regulating signaling pathway (GO:0002764). For the CC terms, enrichment was observed in secretory granule lumen (GO:0034774), cytoplasmic vesicle lumen (GO:0060205), vesicle lumen (GO:0031983), collagen-containing extracellular matrix (GO:0062023), and the external side of the plasma membrane (GO:0009897). Finally, for the MF terms, enrichment was observed for glycosaminoglycan binding (GO:0005539), endopeptidase activity (GO:0004175), serine-type endopeptidase activity (GO:0004252), serine-type peptidase activity (GO:0008236), and serine hydrolase activity (GO:0017171).

KEGG analysis revealed enrichment of crosstalk genes in 565 pathways, predominantly involving the PI3K/Akt signaling pathway, Hippo signaling pathway, and pathways related to proteoglycans in cancer, gastric cancer, and human papillomavirus infection (**Figure 2G**).

### 3.5 Construction of the PPI network and identification of hub genes

**Figure 3:**
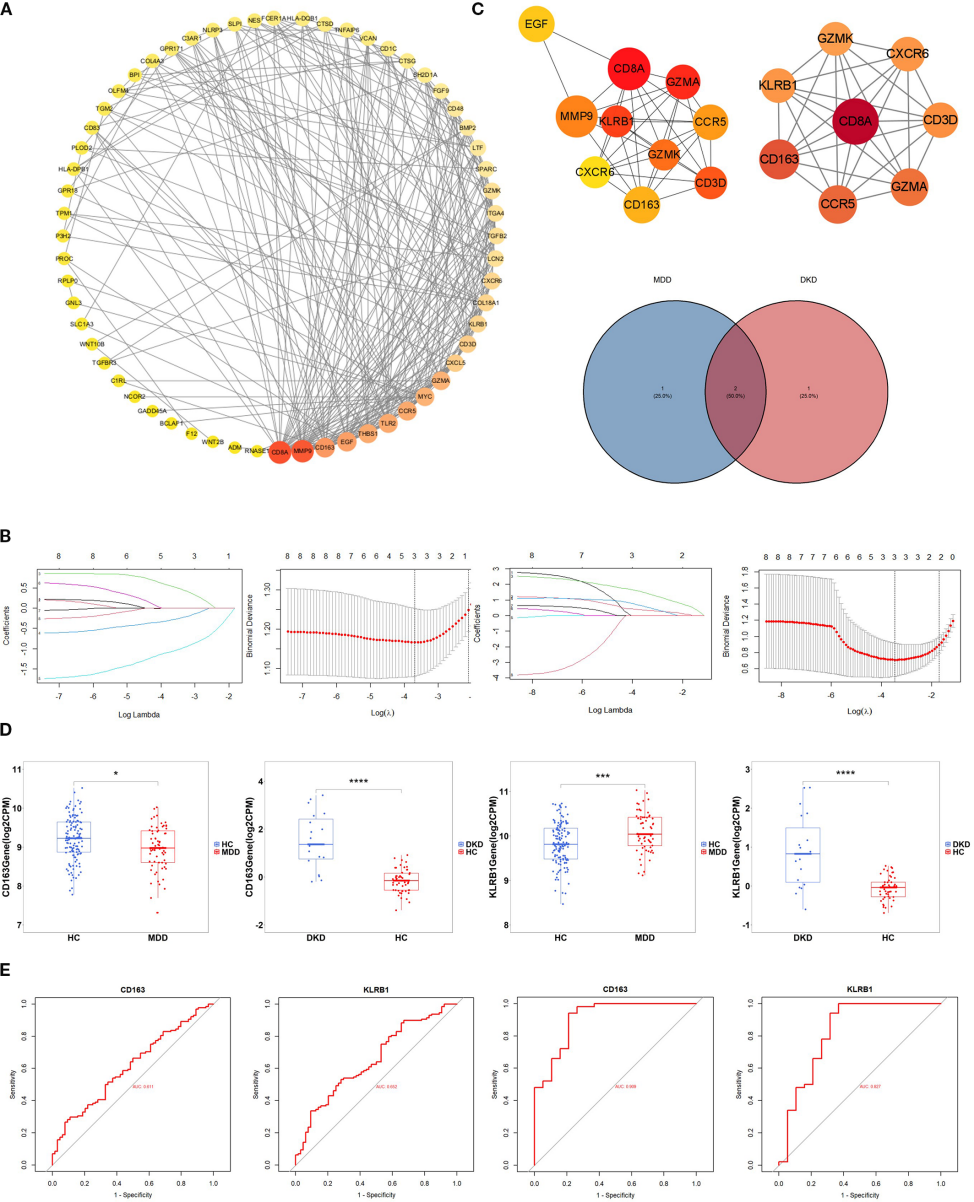
Identification of hub genes. **(A)** PPI network diagram of crosstalk genes. Interaction network of the hub genes identified by Cytohubba.Hub genes extracted by Cytohubba and MCODE. The weight of a hub gene across the network increases with the hue of the gene. **(B)** Distribution of coefficients and coefficient profiles of variables in LASSO regression models in MDD and DKD. **(C)** Venn diagram showing the potential shared diagnostic biomarkers for MDD and DKD. **(D)** Detection of the expression levels of the two potential shared diagnostic biomarkers in MDD and DKD. **(E)** ROC curves of the two potential shared diagnostic biomarkers for MDD (left) and DKD (right). The symbols represent significance levels as follows: * p < 0.05; ** p < 0.01; *** p < 0.001; **** p < 0.0001.

Based on the 83 crosstalk genes, the PPI network created using the STRING database comprised 88 nodes and 316 edges. A network diagram was constructed with Cytoscape software. Cytohubba was utilized to further screen the hub genes, and the top 10 genes were selected based on node degree ranking. In addition, a key module was extracted via the MCODE plugin, and the intersection of the two results (**Figure 3A**) identified the following eight hub genes: CXCR6, GZMA, CD163, KLRB1, GZMK, CCR5, CD3D, and CD8A.

### 3.6 Selection of biomarkers and validation of diagnostic value

LASSO regression analysis was subsequently conducted to identify potential shared diagnostic genes. Three out of eight hub genes were identified in both the GSE98793 dataset and the GSE30122 dataset (**Figure 3B**). Ultimately, two overlapping hub genes, namely, CD163 and KLRB1, emerged as the most promising shared diagnostic biomarkers for both MDD and DKD (**Figure 3C**).

**Figure 3D** illustrates the expression levels of the two diagnostic biomarkers in MDD and DKD. CD163 is upregulated in both diseases, while KLRB1 is upregulated in MDD but downregulated in DKD. Additionally, the sensitivity and specificity of the diagnostic biomarkers were evaluated. In the GSE30122 dataset, both diagnostic biomarkers CD163 (AUC = 0.909) and KLRB1 (AUC = 0.827), demonstrated good diagnostic value. In the GSE98793 dataset, the two biomarkers showed higher diagnostic value (CD163, AUC = 0.611; and KLRB1, AUC = 0.652) (**Figure 3E**). The results showed that both diagnostic biomarkers had significant diagnostic value in disease classification, but the predictive performance in the DKD dataset was better than that in the MDD dataset.

### 3.7 Immune cell infiltration in MDD and DKD

**Figure 4:**
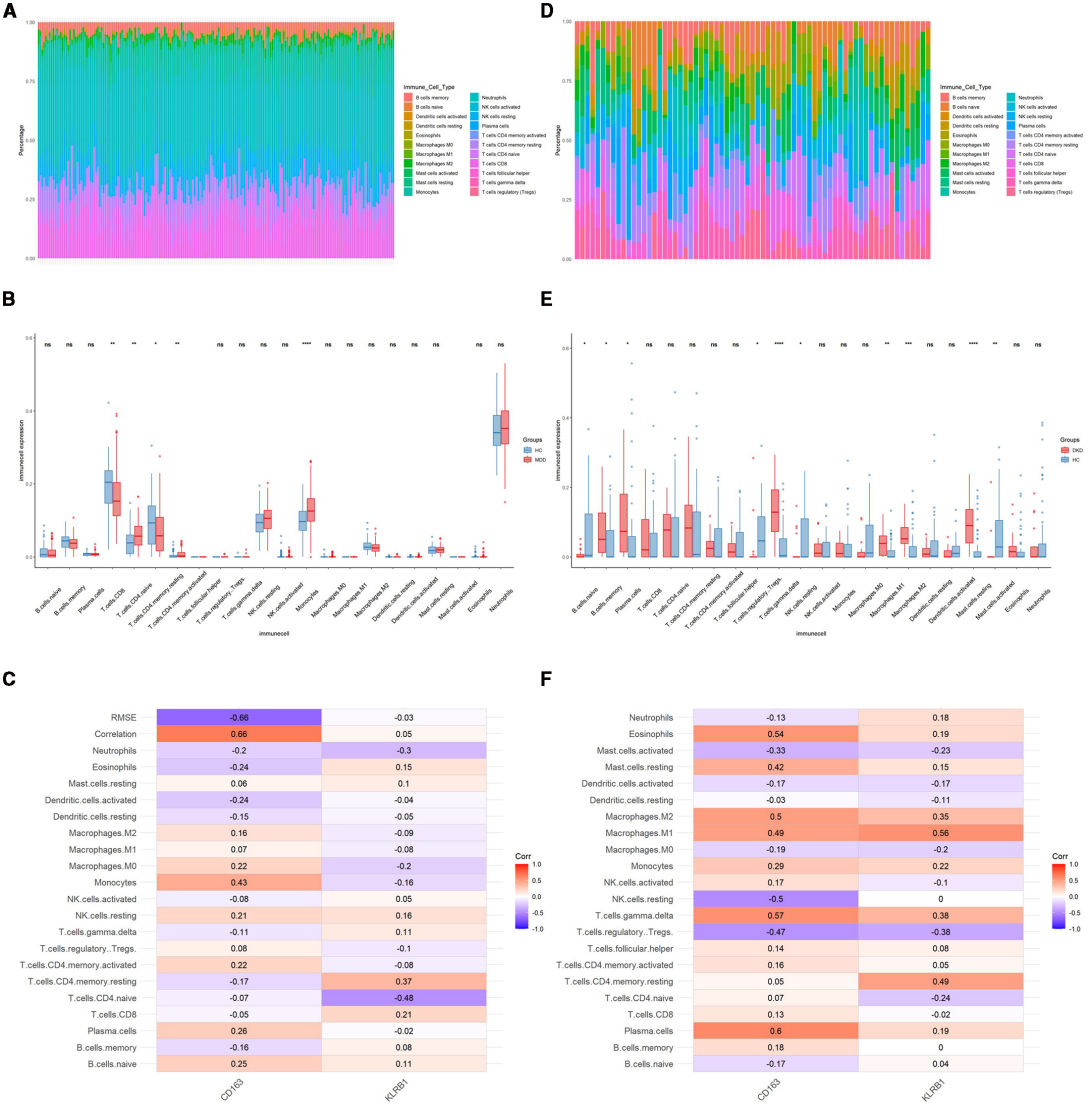
Identification of immune cells in MDD and DKD. **(A)** Immune cell infiltration map in the GSE9879 dataset. **(B)** Box plot showing the comparison of 22 types of immune cells between MDD patients and healthy control individuals. **(C)** Heatmap showing the correlations between common immune cells and potential shared diagnostic biomarkers in the GSE98793 dataset. **(D)** Immune cell infiltration map in the GSE30122 dataset. **(E)** Box plot showing the comparison of 22 types of immune cells between DKD patients and healthy controls. **(F)** Heatmap showing the correlations between common immune cells and potential shared diagnostic biomarkers in the GSE30122 dataset. *p < 0.05; **p < 0.01; ***p < 0.001; ****p < 0.0001; ns, not significant.

To further explore the immune status in MDD and DKD, the percentage of 22 immune cells in each sample was calculated by the CIBERSORT algorithm. **Figures 4A, D** show the infiltration of 22 immune cell types in the GSE98793 and GSE30122 datasets, respectively. In the GSE98793 dataset, only resting CD4+ memory T cells, activated memory CD4+ T cells, and monocytes exhibited significant infiltration in MDD samples (**Figure 4B**). In the GSE30122 dataset, memory B cells, plasma cells, γδ T cells, resting natural killer cells, M1 macrophages, M2 macrophages, and resting mast cells exhibited significant infiltration in DKD (**Figure 4E**). These results suggested that both MDD and DKD patients exhibit immune activation. Although both diseases involve immune activation, the proportions of significantly infiltrating immune cells differed. Additionally, significant correlations were identified for CD163 and KLRB1 expression levels with the infiltration levels of multiple immune cells in both the MDD and DKD samples (**Figures 4C, F**).

### 3.8 Identification of small molecule compounds and molecular docking for MDD and DKD

**Figure 5:**
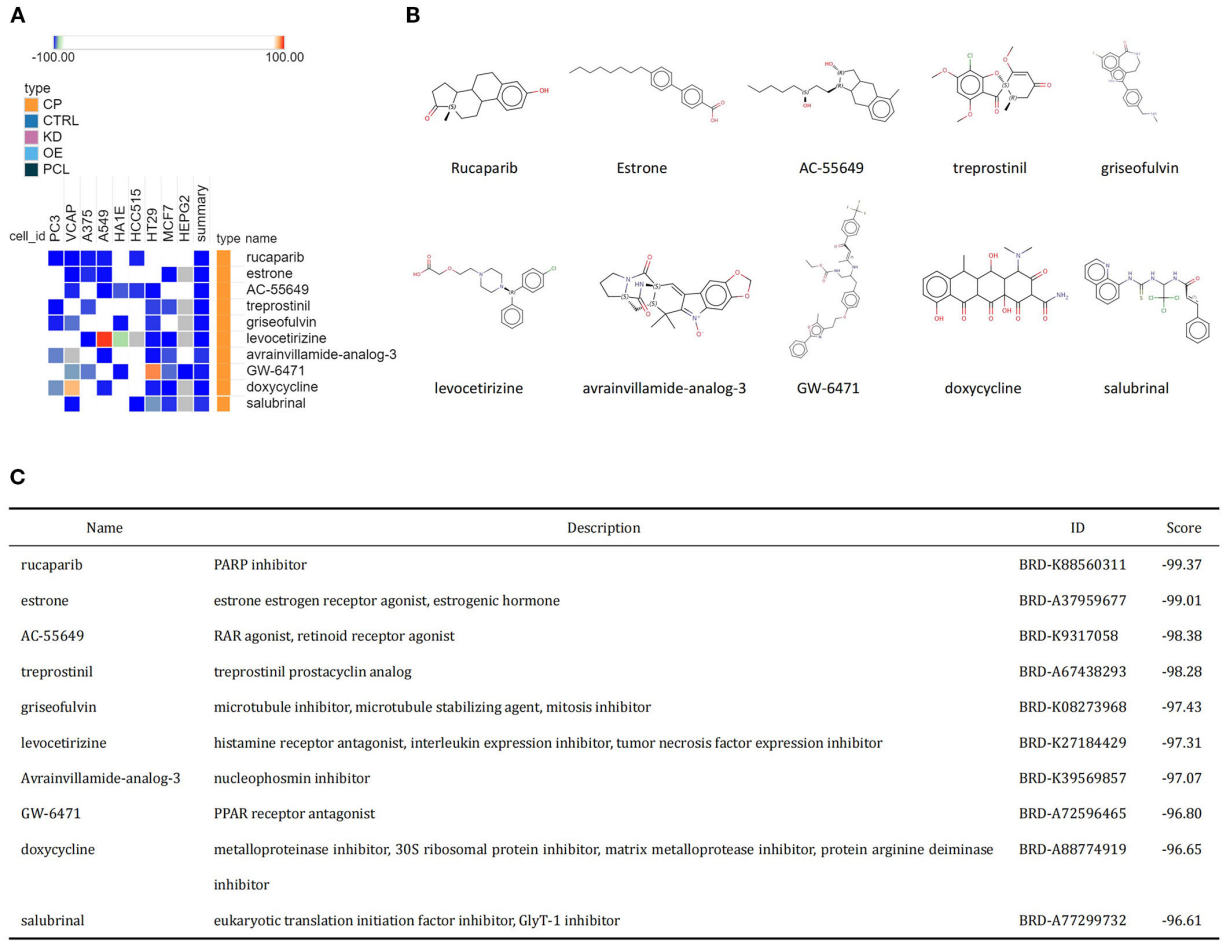
Identification of potential small molecule compounds for the treatment of MDD and DKD via cMAP analysis. **(A)** Heatmap showing the top 10 small molecule compounds with the most significantly negative enrichment scores. **(B)** The chemical structures of the 10 small molecule compounds are shown. **(C)** Descriptions of the top 10 compounds.

The common upregulated crosstalk genes identified in the GSE98793 and GSE30122 datasets were imported into the cMAP database to search for small molecule compounds capable of reversing the expression of pathogenic genes associated with MDD and DKD. The top 10 compounds with the highest negative scores included rucaparib, estrone, AC-55649, treprostinil, griseofulvin, levocetirizine, avrainvillamide-analog-3, GW-6471, doxycycline, and salubrinal, which are considered potential therapeutic agents (**Figure 5A**). The targeting pathways and chemical structures of these 10 compounds are shown in **Figures 5B, C**.

**Figure 6:**
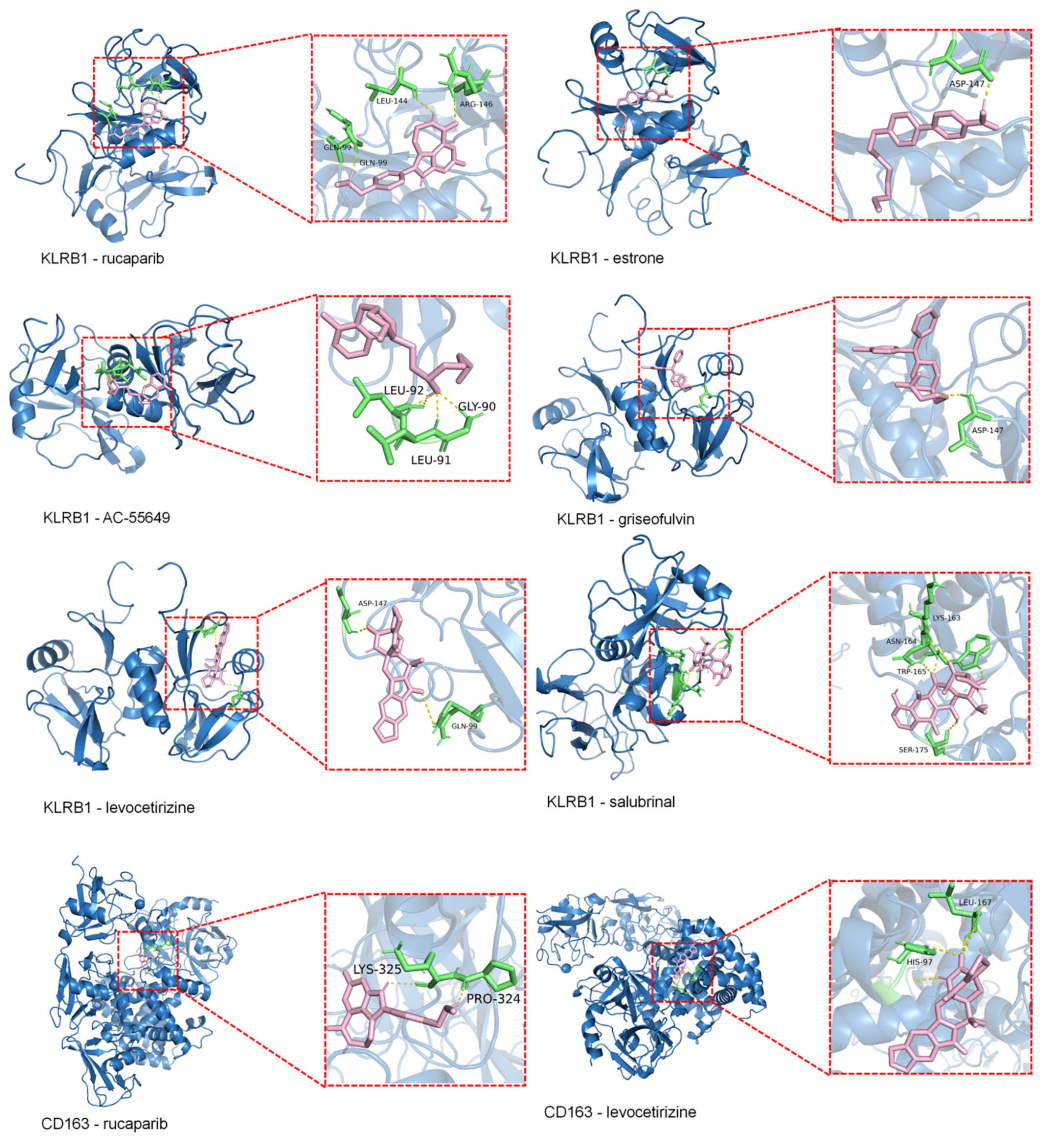
Molecular docking of KLRB1 and CD36 with small molecules (Binding Energy > –5 kcal/mol). Blue represents the macromolecular protein structure, green indicates the key amino acid residues involved in binding, pink denotes the small-molecule ligands, and yellow lines highlight the hydrogen bonds formed between the ligands and the protein residues.

Molecular docking results showed that the binding energies of CD163 with rucaparib and levocetirizine were –6.26 and –6.60 kcal/mol, respectively. KLRB1 exhibited binding energies lower than –5.00 kcal/mol with rucaparib, estrone, AC-55649, griseofulvin, levocetirizine, and salubrinal, with the lowest binding energy observed for levocetirizine at –6.09 kcal/mol (**Figure 6**). Detailed information on the binding energies, key binding sites, and number of hydrogen bonds between each small molecule and target protein is provided in **Supplementary Table 4**.

## 4 Discussion

Previous studies have demonstrated a bidirectional association between MDD and DM, with Type 2 DM being linked to a 24% higher prevalence of MDD, and MDD showing a 60% higher incidence in individuals with Type 2 DM (24, 25). Research on the comorbidity of DM and MDD has also been increasing annually. Fang et al. confirmed a mutually influential relationship between MDD and DKD (11). The pathogenesis of MDD is associated with immune system dysregulation, and enhanced expression of inflammatory markers (IL-6, C-reactive protein, and TNF-α) increases the risk of DKD (26, 27). In addition, MDD increases the activity of the hypothalamic-pituitary-adrenal axis, sympathetic nervous system, stress hormones, and cortisol. These factors stimulate glucose production, lipolysis, and free fatty acid cycling but decrease insulin secretion and sensitivity, potentially resulting in an elevated risk of developing DKD (28). Although the bidirectional relationship between DKD and MDD has been acknowledged, the underlying mechanisms remain poorly understood. Our cross-sectional analysis revealed a significant association between DKD and MDD, and genetic analyses indicated a degree of genetic correlation between the two conditions. However, there was no clear evidence supporting a causal relationship, which may be influenced by unmeasured confounders such as lifestyle or medication factors. These findings suggest a potential shared genetic background rather than a direct causal pathway, underscoring the complexity of the relationship. Therefore, we hypothesized that DKD and MDD share common differential genes and biological pathways, which we proceeded to investigate.

The present study identified 83 genes associated with MDD and DKD through differential expression analysis of the GSE98793 and GSE30122 datasets. Enrichment analysis discovered a significant presence of the PI3K/Akt signaling pathway and Hippo signaling pathway in MDD and DKD. Eight hub genes, namely, CXCR6, GZMA, CD163, KLRB1, GZMK, CCR5, CD3D, and CD8A, were identified from the PPI network, all of which are closely related to MDD and DKD. In addition, LASSO analysis was used to identify two hub genes, namely, CD163 and KLRB1, as potential shared diagnostic biomarkers, and their diagnostic value was validated, confirming their diagnostic significance in both diseases. Immune infiltration analysis using the DEGs of MDD and DKD was performed. T cells and monocytes exhibited significant infiltration in MDD, while various immune cells, including B cells, macrophages, and mast cells, exhibited significant infiltration in DKD. Finally, drug prediction was performed on the genes involved in the crosstalk between MDD and DKD, identifying multiple small molecule compounds as potential therapeutic drugs for MDD and DKD.

Enrichment analysis revealed that the comorbidity of MDD with DKD was primarily associated with the PI3K/Akt and Hippo signaling pathways. Additionally, the comorbidity of MDD with DKD was closely related to various cancers, such as bladder cancer, gastric cancer, and breast cancer, as well as autoimmune diseases, such as rheumatoid arthritis. Thus, the comorbidity of MDD and DKD may share common biological mechanisms with certain cancers. The abnormal activation of these two signaling pathways in MDD and DKD may be linked to an elevated risk of cancer, indicating a potential interplay or cross-impact between them. The PI3K/Akt signaling pathway is a critical cellular signaling cascade that is pivotal for numerous biological processes, including cell survival, proliferation, differentiation, and metabolism (29). It has been suggested that the activity of the PI3K/AKT pathway may be inhibited in diabetic states, which can lead to a series of pathophysiological alterations, including increased cell apoptosis, enhanced oxidative stress, cell proliferation, and inflammatory responses (30). Further study of the mechanism of the PI3K/AKT pathway in DKD will enhance the understanding of the disease pathogenesis and lay a theoretical groundwork for the development of novel therapeutic strategies. mTOR is a key downstream effector of the PI3K/AKT signaling pathway and plays a central role in regulating cell growth, metabolism, and autophagy. Abnormal activation or inhibition of mTOR is closely associated with the development of various diseases. In Alzheimer’s disease, excessive activation of mTOR may inhibit autophagy, leading to the accumulation of abnormal proteins, which is thought to contribute to the disease’s pathogenesis (31). In contrast, studies suggest that in patients with major depressive disorder, mTOR signaling activity may be suppressed, and activation of this pathway has been associated with antidepressant effects (32). Lima et al. reported that valproic acid (VPA) has antidepressant effects, which may be associated with the modulation of the PI3K/Akt/mTOR signaling pathway (33). The present findings provide important insight for exploring new targets for the treatment of MDD. Additionally, the present study provides a theoretical basis for the clinical application of PI3K/Akt/mTOR pathway modulators as antidepressant medications.

The Hippo pathway regulates cell proliferation, apoptosis, and organ size, thereby maintaining tissue and organ homeostasis, and the main components of the Hippo pathway include MST1/2, LATS1/2, and their substrates. The Hippo pathway was initially discovered in Drosophila and has since been extensively studied in mammals (34). The Hippo pathway is an important cellular signaling pathway that may contribute to the development and progression of DKD, and related studies are ongoing. As a downstream effector of the Hippo signaling pathway, YAP promotes renal interstitial fibrosis in DKD, and high expression of YAP is correlated with increased systolic blood pressure, blood urea nitrogen, and creatinine, as well as with the progression of DKD staging and DKD pathological classification. Inhibiting YAP activity may slow the progression of DKD (34, 35). Therefore, targeting the Hippo signaling pathway may be a therapeutic strategy for DKD. There is no conclusive evidence linking the Hippo pathway to MDD occurrence and progression. However, previous studies on immunological characteristics have revealed the involvement of MST1/2 in regulating lymphocyte adhesion, migration, and CD4+ antigen recognition (36), which aligns with the present immune infiltration analysis, demonstrating that CD4+ T cells were significantly infiltrated in MDD, suggesting that the Hippo pathway may have a potential biological link to MDD. Currently, direct clinical and experimental data confirming the association between MDD and the Hippo signaling pathway are lacking. Thus, additional research is warranted to elucidate the mechanisms of the Hippo signaling pathway in MDD and its potential value as a therapeutic target for MDD.

Through the PPI network, eight hub genes that are closely associated with the immune system and inflammation regulation were identified. Among them, CD163, CCR5, CD3D, KLRB1, and CD8A serve as surface markers of immune cells and play a role in regulating immune responses. GZMA and GZMK encode proteases found in natural killer cells that are involved in cytotoxicity and the modulation of inflammatory responses (37). CXCR6, also known as CD186, is a chemokine receptor that is mainly expressed in immune cells, especially in activated T cells, natural killer cells, macrophages, and dendritic cells. CXCR6 participates in the immune response within the body, the inflammatory response, tissue cell migration, and tumor immunity (38). These eight hub genes share commonalities in regulating the immune system and inflammation, suggesting their pivotal roles in the pathogenesis of both MDD and DKD. These shared characteristics may explain their identification as relevant genes in both MDD and DKD.

Among the hub genes, CD163 and KLRB1 were identified as potential shared diagnostic biomarkers for MDD and DKD according to LASSO analysis. On the surface of macrophages, CD163 is a widely expressed receptor protein that serves as a marker for monocytes and tissue macrophages. CD163 participates in immune regulation by binding and clearing hemoglobin, regulating cytokine production and release, and modulating inflammatory responses. Furthermore, changes in serum CD163 levels are closely associated with disease status and inflammation severity, suggesting that CD163 is a potential biomarker of inflammation for disease diagnosis and monitoring (39). Research has shown that in patients with DM, the CD163 expression level in monocytes is negatively correlated with the type and severity of diabetic complications (40). However, another study has indicated that glomerular CD163+ macrophages are positively associated with DKD grade, interstitial fibrosis, tubular atrophy, and glomerulosclerosis (41). In 2017, Samuelsson et al. confirmed that CD163 is a promising early diagnostic biomarker for DKD (42). Similarly, Wang et al. corroborated this finding, aligning with the results of the present study (43).

KLRB1, also known as CD161, is a cell surface molecule belonging to the C-type lectin receptor family. KLRB1 is a transmembrane protein widely expressed in humans and other mammals. KLRB1 plays a pivotal regulatory role in the immune system and is particularly associated with natural killer (NK) cells and certain subsets of T cells (44). Currently, there are few studies on the association of KLRB1 with MDD and DKD, but its possible involvement in the immune system to regulate biological processes, such as the inflammatory response, autoimmune diseases, and antitumor immunity, may be relevant to the development of MDD and DKD. Therefore, further investigation of KLRB1 may aid in enhancing the understanding of the regulatory mechanisms of the immune system and provide new targets and strategies for the treatment of both diseases.

In addition, CD163 and KLRB1 are closely associated with tumor development. CD163 macrophages are abundant in the tumor microenvironment, and CD163 has been utilized for identifying tumor-associated macrophages in malignant diseases. For example, increased numbers of CD163+ macrophages and CD163+ gastric cancer cells are correlated with gastric tumor invasion and poor prognosis (45). Cheng et al. explored the relationship between KLRB1 and pancancer, and they reported that KLRB1 may impact tumor immunity by modulating the levels of infiltrating immune cells, particularly macrophages and lymphocytes, and that KLRB1 acts as a protective gene in the majority of cancers (46). The enrichment analysis in the present study revealed that MDD and DKD were closely related to various tumors, which may be associated with the molecular mechanisms of CD163 and KLRB1. Future studies will explore the mechanism of CD163 and KLRB1 in patients with DKD combined with MDD to offer novel insights into the clinical diagnosis and treatment of this disease.

As mentioned above, the immune mechanisms of MDD and DKD are pivotal in the onset and progression of these diseases. An increased inflammatory response, activation of immune cells, and neuroimmune interactions may be common immune mechanisms in both diseases. In MDD patients, immune cells, such as T cells, macrophages, and monocytes, may be in an activated state, and their number and activity may increase (47). In contrast, in DKD patients, immune cells, such as macrophages, dendritic cells, lymphocytes, mast cells, and neutrophils, are involved in the genesis and development of DKD (48). Immune cells produce various inflammatory factors, such as interleukin-1β (IL-1β), tumor necrosis factor-α (TNF-α), and interleukin-6 (IL-6). Moreover, abnormal production of these inflammatory factors may affect neuronal activity, neurotransmitter levels, and neuroplasticity, leading to depressive symptoms (49). Moreover, the aforementioned inflammatory factors have been shown to exert a pivotal influence on DKD (48). The immune cell infiltration analysis findings in the present study align with previous research findings. CD4+ T cells and monocytes significantly infiltrated MDD patients, while various immune cells, such as B cells, macrophages, and mast cells, significantly infiltrated DKD patients. The two potential diagnostic biomarkers identified in the present study, namely, CD163 and KLRB1, are widely expressed in various immune cells, such as monocytes, macrophages, and lymphocytes, and they contribute to the pathogenesis and progression of diseases by modulating inflammation and immune responses.

In the present study, 12 commonly upregulated crosstalk genes in MDD and DKD were imported into the cMAP database, which identified 10 small molecule compounds (rucaparib, estrone, AC-55649, treprostinil, griseofulvin, levocetirizine, avrainvillamide-analog-3, GW-6471, doxycycline, and salubrinal) as potential therapeutic agents. cMAP analysis revealed that rucaparib had the most significant negative enrichment score, indicating that it effectively influences the expression of pathogenic genes associated with the comorbidity of MDD and DKD. Rucaparib is a poly (ADP-ribose) polymerase (PARP) inhibitor primarily used for the treatment of metastatic breast cancer patients with BRCA1 or BRCA2 mutations. Studies have also shown the inhibitory effect of rucaparib on diseases, such as ovarian cancer and prostate cancer (50). There are no definitive studies demonstrating a role for rucaparib in DKD or MDD. However, evidence suggests that PARP is involved in inflammation and metabolic regulation. Overactivation of PARP may lead to pathophysiological processes, such as excessive increases in the inflammatory response, apoptosis, and metabolic abnormalities (51). PARP inhibitors have the potential to alleviate inflammation and metabolic disorders by inhibiting PARP activity. PARP inhibitors have been demonstrated to significantly reduce the development of nephropathy caused by DM, as well as reduce oxidative stress levels, inhibit inflammatory responses, and alleviate renal fibrosis (52). In addition, the expression level of PARP1 is significantly elevated in MDD patients, and it decreases after electroconvulsive therapy (53). Therefore, PARP inhibitors have a theoretical basis for the treatment of DKD combined with MDD, and they may become a potential strategy for disease treatment. Additionally, other small molecules, such as histamine receptor inhibitors, interleukin expression inhibitors, NPM1 protein inhibitors, and PPAR receptor antagonists, are closely related to inflammation regulation. These compounds may hold promise for the treatment of MDD, DKD, and other inflammation-related diseases.

The present study had several limitations. Although CD163 and KLRB1 were identified as diagnostic biomarkers using bioinformatics methods, the lack of comprehensive validation and analysis of clinical samples may affect their reliability in clinical applications, thus requiring further experimental support. Additionally, enrichment analysis and drug prediction were based solely on gene expression data analysis, and further experimental validation and functional studies are required to confirm the biological significance and mechanism of these findings. Furthermore, due to the lack of lifetime diagnostic information and diabetes subtyping in the NHANES database, our definitions of MDD and diabetes have certain limitations, which should be addressed in future studies by incorporating clinical classifications and expert consultation. Finally, the NHANES dataset does not provide explicit information on type 1 and type 2 diabetes, which limits our ability to conduct subtype-specific analyses; future studies are encouraged to incorporate clinical classification for greater precision.

## 5 Conclusion

The present study indicated that CD163 and KLRB1 are potential shared diagnostic biomarkers for MDD and DKD, and it revealed the underlying biological processes common to both diseases. These findings provide important clues for future studies and are expected to provide new targets and strategies for the diagnosis and treatment of MDD and DKD.

## Data Availability

The original contributions presented in the study are included in the article/**Supplementary Material**, further inquiries can be directed to the corresponding authors.
