# Supplementary material for: Exploring the relationship and shared mechanisms of major depressive disorder and diabetic kidney disease: a comprehensive clinical and genetic analysis
Source: Front Psychiatry. 2025 Sep 29;16:1546733. doi: 10.3389/fpsyt.2025.1546733 (PMC12515961; doi:10.3389/fpsyt.2025.1546733)
Supplement: Supplementary file 1 [file DataSheet1.docx]

Supplementary Material

# Supplementary Tables

#

**Supplementary Table 1.** Overview of the GWAS data used in the study.

| **Phenotypes** | **PMID** | **Population** | **Sample size** | **Pvalue** | **Number of SNPs** | **LD** | **F-statistic range** |
| --- | --- | --- | --- | --- | --- | --- | --- |
| MDD | 37464041 | European | 500,199 | 5.00E-08 | 157 | 0.001 | 29.6 - 96.59 |
| DKD | 37219871 | European | 435,971 | 5.00E-06 | 10 | 0.001 | 21.04 - 42.18 |

**Supplementary Table 2.** Genetic correlation estimates from LDSC regression.

| **Phenotype 1 (h2)** | **Phenotype 2 (h2)** | **rg** | **SE** | **P value** |
| --- | --- | --- | --- | --- |
| DKD （0.0026） | MDD （0.0454） | 0.2153 | 0.0814 | 0.0082 |

**Supplementary Table 3.** Directional pleiotropy test and heterogeneity test for the causal associations between MDD and DKD.

| **Exposures** | **Outcomes** | **Heterogeneity test** | | | | **Pleiotropy test** | | **MR-PRESSO** |
| --- | --- | --- | --- | --- | --- | --- | --- | --- |
|  |  | **MR-Egger** | | **IVW** | | **MR-Egger intercept** | | **Global test P value** |
|  |  | **Q** | **pval** | **Q** | **pval** | **Intercept** | **P** |  |
| MDD | DKD | 139.021 | 0.126 | 139.448 | 0.134 | -0.017 | 0.543 | 0.210 |
| DKD | MDD | 4.880 | 0.559 | 9.558 | 0.215 | 0.014 | 0.074 | 0.223 |

**Supplementary Table 4.** Summary of molecular docking results between small molecules and hub gene proteins.

| **Molecule Name** | **Target Protein  (Hub Gene)** | **Binding Energy  (kcal/mol)** | **Interacting Residues** | **Number of Hydrogen Bonds** |
| --- | --- | --- | --- | --- |
| rucaparib | CD163 | -6.26 | LYS-325, PRO-324 | 2 |
| estrone | CD163 | -4.87 | ARG-311, GLU-314,  LYS-325 | 3 |
| AC-55649 | CD163 | -4.36 | ASN-213, TRP-350 | 2 |
| treprostinil | CD163 | -4.66 | LYS-325 | 1 |
| griseofulvin | CD163 | -4.63 | LYS-170 | 1 |
| levocetirizine | CD163 | -6.60 | HIS-97, LEU-167 | 3 |
| avrainvillamide -analog-3 | CD163 | -2.99 | MET-159 | 1 |
| GW-6471 | CD163 | -3.84 | ALA-212, TYR-242 | 2 |
| doxycycline | CD163 | -3.59 | MET-159 | 1 |
| salubrinal | CD163 | -4.99 | ASP-94, PHE-290,  LYS-291 | 5 |
| rucaparib | KLRB1 | -5.49 | GLN-99, LEU-144,  ARG-146 | 3 |
| estrone | KLRB2 | -5.20 | ASP-147 | 1 |
| AC-55649 | KLRB3 | -5.92 | LEU-92, GLY-90,  LEU-91 | 4 |
| treprostinil | KLRB4 | -4.70 | ASP-147 | 1 |
| griseofulvin | KLRB5 | -5.38 | ASP-147 | 1 |
| levocetirizine | KLRB6 | -6.09 | GLN-99, ASP-147 | 2 |
| avrainvillamide -analog-3 | KLRB7 | -3.26 | NA | 0 |
| GW-6471 | KLRB8 | -4.80 | GLN-99 | 1 |
| doxycycline | KLRB9 | -4.98 | GLN-100, LEU-91,  LEU-92 | 4 |
| salubrinal | KLRB10 | -5.34 | LYS-163, ASN-164,  TRP-165, SER-175 | 5 |

# Supplementary Figures


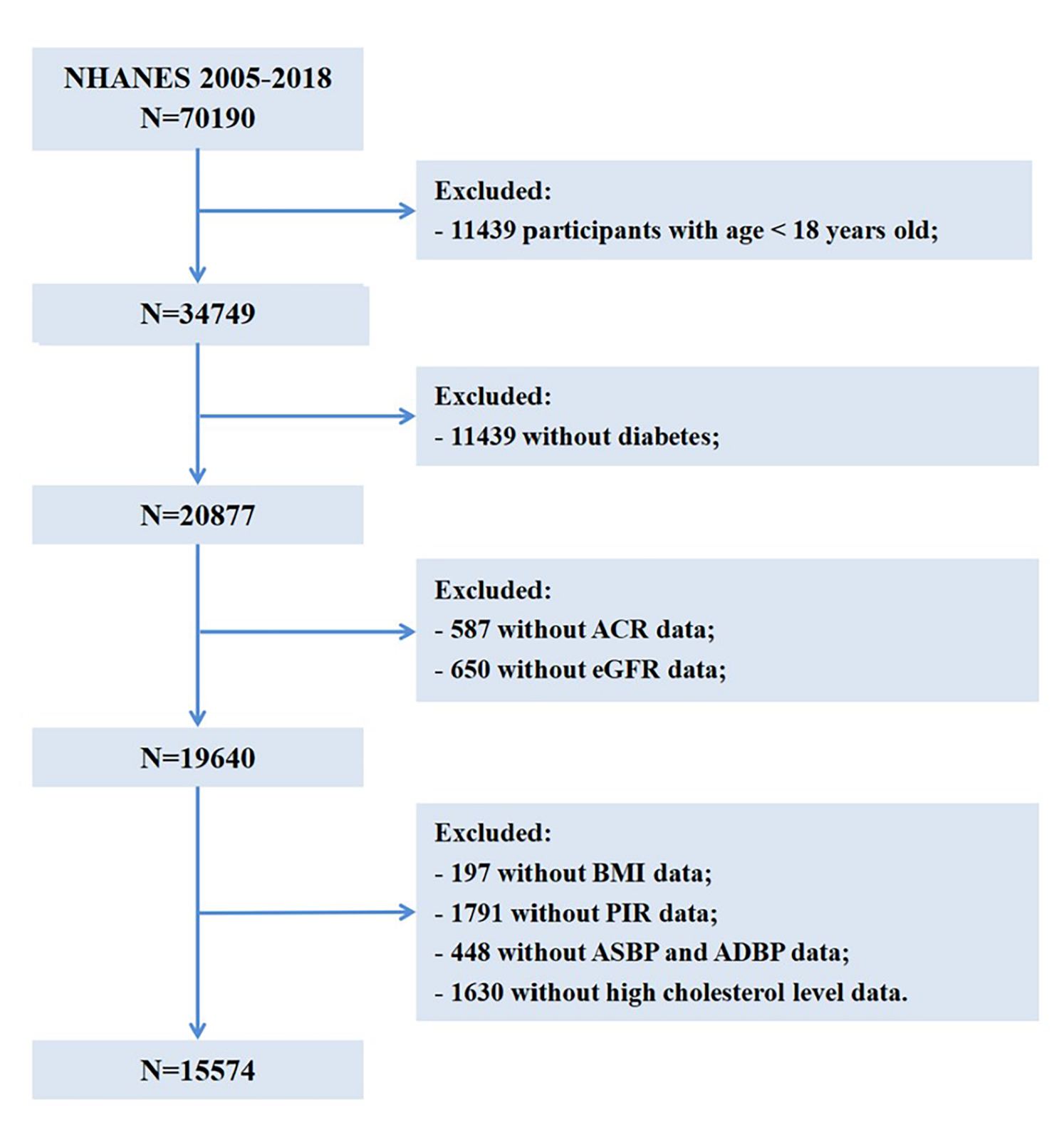


**Supplementary Figure 1. A flowchart showing the selection of study participants.**

Abbreviations: NHANES - National Health and Nutrition Examination Survey; ACR - albumin-to-creatinine ratio; eGFR–Estimated Glomerular Filtration Rate; PIR - poverty income ratio; BMI - body mass index; SBP - Systolic Blood Pressure; DBP - Diastolic Blood Pressure.


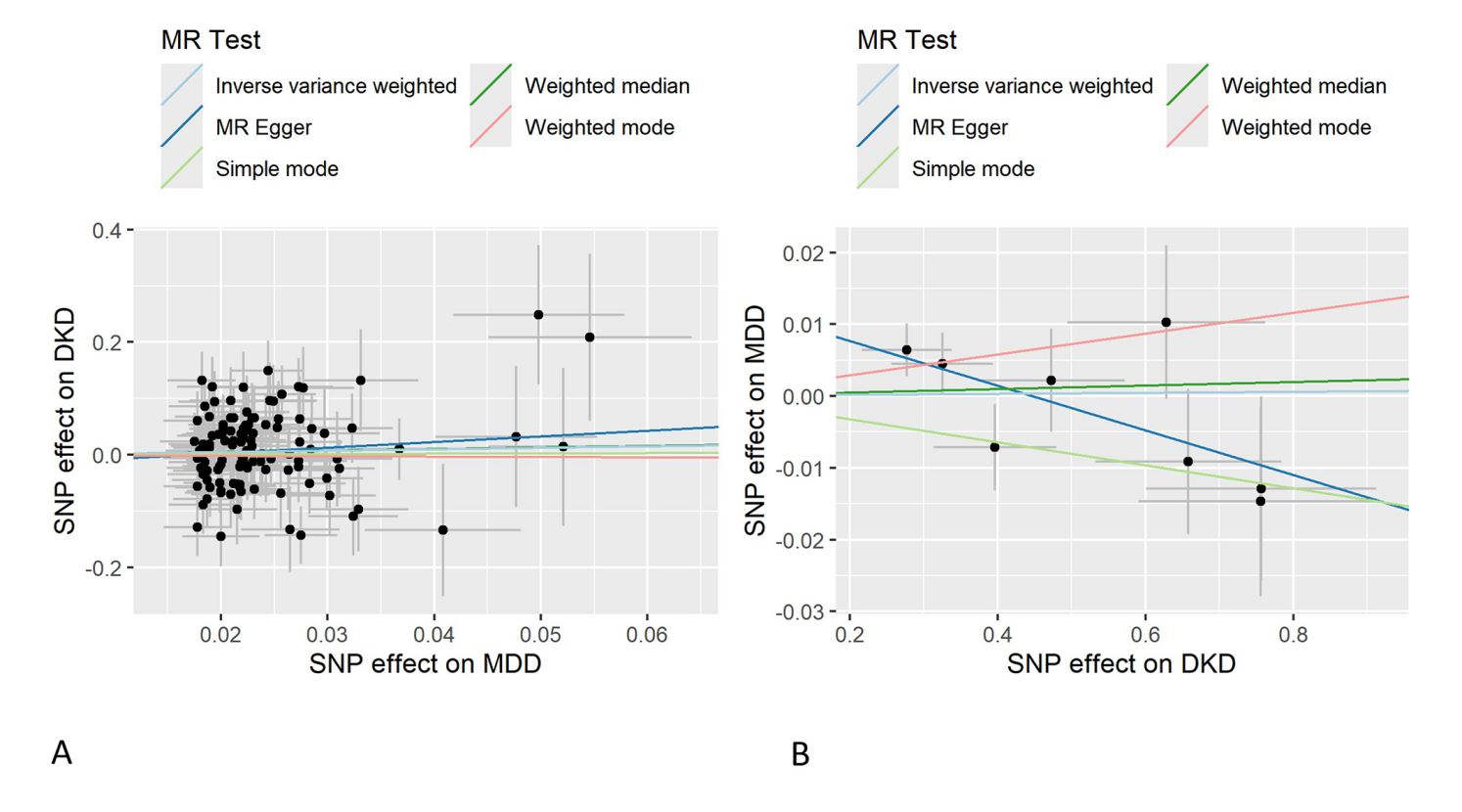


**Supplementary Figure 2. Scatter plots of genetic correlation by different MR analysis methods** (A) Scatter plot of genetic correlation between MDD and DKD. (B) Scatter plot of genetic correlation between DKD and MDD.
